# Characterization of three new mitochondrial genomes of Coraciiformes (*Megaceryle lugubris*, *Alcedo atthis*, *Halcyon smyrnensis*) and insights into their phylogenetics

**DOI:** 10.1590/1678-4685-GMB-2019-0392

**Published:** 2020-10-05

**Authors:** Meidong Jing, Huanhuan Yang, Kai Li, Ling Huang

**Affiliations:** 1 Nantong University, School of Life Sciences, Nantong, Jiangsu, P. R. China.; 2 Ludong University, School of Life Sciences, Yantai, Shandong, P. R. China.; 3 Nantong Xingdong International Airport, Nantong, Jiangsu, P. R. China.

**Keywords:** Mitogenome, Alcedo atthis, Halcyon smyrnensis, Megaceryle lugubris, phylogeny

## Abstract

Coraciiformes contains more than 200 species with great differences on external morphology and life-style. The evolutionary relationships within Coraciiformes and the phylogenetic placement of Coraciiformes in Aves are still questioned. Mitochondrial genome (mitogenome) sequences are popular markers in molecular phylogenetic studies of birds. This study presented the genome characteristics of three new mitogenomes in Coraciiformes and explored the phylogenetic relationships among Coraciiformes and other five related orders with mitogenome data of 30 species. The sizes of three mitogenomes were 17,383 bp (*Alcedo atthis*), 17,892 bp (*Halcyon smyrnensis*) and 17,223 bp (*Megaceryle lugubris*). Each mitogenome contained one control region and 37 genes that were common in vertebrate mitogenomes. The organization of three mitogenomes was identical to the putative ancestral gene order in Aves. Among 13 available Coraciiform mitogenomes, 12 protein coding genes showed indications of negative selection, while the MT-ND6 presented sign of positive selection or relaxed purifying selection. The phylogenetic results supported that Upupidae and Bucerotidae should be separated from Coraciiformes, and that Coraciiformes is more closely related to Piciformes than to Strigiformes, Trogoniformes and Cuculiformes. Our study provide valuable data for further phylogenetic investigation of Coraciiformes.

## Introduction

Coraciiformes consists of more than 200 species with great differences in distribution region, body size, external morphology and life-style. There are 25 Coraciiform species in China, among which the largest species (*Buceros bicornis*) is about three to four kilograms in weight and 119 to1 28 centimeters in body length. However, the smallest species (*Alcedo atthis*) is only about 30 to 40 grams in weight and 150 to 170 millimeters in length (Zhao, 2001). The habitat and diet of Coraciiform species are also diverse. Some species (Alcedinidae) live around rivers and lakes, and feed on fish and shrimps; some species (Bucerotidae, Meropidae, Upupidae) live in forests and feed on fruits, seeds or insects; other species (Coraciidae) live in the plain and feed on insects and small animals (Zhao, 2001).

In classical taxonomy, Coraciiformes included 10 families (Alcedinidae, Bucerotidae, Brachypteracidae, Coraciidae, Leptosomatidae, Meropidae, Momotidae, Phoeniculidae, Todidae, and Upupidae) (Wetmore, 1960). However, later investigations based on anatomy of the feeding apparatus (Burton, 1984) and fossil records (Olson, 1985) proposed that the “Bucerotes” (Bucerotidae, Phoeniculidae and Upupidae) should be separated from Coraciiformes. The phylogenetic position of Leptosomatidae also should be questioned according to the analyses of different sets of morphological characters (Mayr *et al.*, 2003; Mayr, 2005). In addition, different morphological assessments deduced inconsistent conclusions on the relationships among Coraciiformes and other related orders. For example, studies based on myological characters showed that Trogonidae (Trogoniformes) should be included in Coraciiformes (Cracraft, 1981; Maurer and Raikow, 1981), while investigations with osteological characters suggested a distant relationship between Trogonidae and Coraciiformes (Höfling and Alvarenga, 2001). Studies by Burton (1984) and Olson (1985) proposed that “Bucerotes” was more closely related to Piciformes than to other families in Coraciiformes, while analyses of 98 morphological characters suggested a closer relationship between Piciformes and classical Coraciiformes (Mayr, 2005).

Molecular phylogenetic investigations based on nuclear (Sibley and Ahlquist, 1990; Kemp, 1995; Johansson *et al.*, 2001; Hackett *et al.*, 2008; Jetz *et al.*, 2012; Ödeen and Hästad, 2013; Jarvis *et al.*, 2014; Prum *et al.*, 2015; Reddy *et al.*, 2017) or mitochondrial gene markers (de los Monteros, 2000; Ericson *et al.*, 2006; Pacheco *et al.*, 2011; Mahmood *et al.*, 2014; Sun *et al.*, 2017; Tamashiro *et al.*, 2019) provided powerful evidence on the separated position of “Bucerotes”*.* The separated position of Leptosomatidae was also supported by molecular phylogenetic investigations (Ericson *et al.*, 2006; Hackett *et al.*, 2008). However, most of above studies aimed to resolve the high-level phylogenetic relationships in Aves, species involved in these studies were limited, and the relationships among Coraciiformes and related orders were still in controversy. As for the relationship among Coraciiformes, “Bucerotes”, Piciformes and Trogoniformes, some analyses using multiple nuclear genes (e.g., Hackett *et al.*, 2008; Jetz *et al.*, 2012; Ödeen and Hästad, 2013; Jarvis *et al.*, 2014; Prum *et al.*, 2015; Reddy *et al.*, 2017) and mitogenome data (e.g., Mahmood *et al.*, 2014; Tamashiro *et al.*, 2019) supported the topology of (Trogoniformes, (Bucerotes, (Coraciiformes, Piciformes))), while some studies with mtDNA data indicated the topology of ((Coraciiformes, Bucerotes), (Piciformes, Trogoniformes))” (e.g. Pacheco *et al.*, 2011) or (Piciformes, (Coraciiformes, (Trogoniformes, Bucerotes))) (de los Monteros, 2000).[Bibr B27]

Mitochondrial genomes (mitogenomes), because of their advantageous characteristics (small size, simple organization, lack of recombination, rapid nucleotide substitution), have been extensively applied in phylogenetic studies of birds since the report of chicken mitogenome (e.g. Desjardins *et al.*, 1990; Cooper *et al.*, 2001; Paton *et al.*, 2002; Morgan-Richards *et al.*, 2008; Pratt *et al.*, 2009; Nabholz *et al.*, 2010; Pacheco *et al.*, 2011; Zhou *et al.*, 2014; Bruxaux *et al.*, 2018; Tamashiro *et al.*, 2019). Some researchers proposed that the molecular studies using only mtDNA markers have limitations in testing phylogenetic hypotheses, because incomplete lineage sorting, adaptive introgression, demographic disparities or sex-biased asymmetries exist in many animal systems (Toews and Brelsford, 2012). However, phylogenies inferred from mitogenome data can complement and confirm the results based on nuclear gene markers, they are still essential in phylogenetic investigations. Up to date, mitogenomes of about 800 avian species have been released in GenBank, among which only 13 species belong to Coraciiformes (three species in this study were included). The accumulation of mitogenome data in Coraciiformes will be helpful to explore the phylogenetic puzzles on this order.

Here, we provide three new mitogenome data in Alcedinidae of Coraciiformes: Common Kingfisher (*Alcedo atthis*), White-throated Kingfisher (*Halcyon smyrnensis*) and Crested Kingfisher (*Megaceryle lugubris*). This study aims to 1) elucidate the structural characteristics of three mitogenomes and compare our data with other Coraciiform mitogenomes available in GenBank; 2) explore the phylogenetic relationships among 12 families of six orders (Coraciiformes, Piciformes, Strigiformes, Cuculiformes, Trogoniformes, Psittaciformes) with mitogenome data of 30 species.

## Material and Methods

### Genomic DNA preparation

The samples of *Megaceryle lugubris*, *Alcedo atthis* and *Halcyon smyrnensis* were collected from Nantong national airport, Jiangsu Province, China. The identification of the specimens was according to external morphologies (Sibley and Monroe, 1990). The muscle tissues were preserved in absolute ethanol and were stored at −80 °C. A Wizard Genomic DNA purification kit (Promega, Madison, WI, USA) was used to extract the total genomic DNA. Concentration of the genomic DNA was determined with a spectrophotometer and was adjusted to 50 ng/μL.

### PCR amplification and sequencing

To amplify overlapping segments spanning the whole mitogenome, 28 sets of primers reported by Sorenson *et al.* (1999) were used. The amplified segments were all smaller than 1,500 bp, and all segments overlapped each other by 200 bp. The amplifications were completed in a Mycycler Gradient thermocycler (Bio-Rad), and the volume of each reaction was about 50 μL, containing 25 μL of Premix Taq (TaKaRa TaqTM Version 2.0 plus dye, Takara Biotechnology, Dalian, China), 1 μL (20 μM) of each primer, 22.5 μL of deionized water and 0.5 μL of genomic DNA (about 25-30 ng). The PCR processes were consistent with those reported previously (Sun *et al.*, 2017). To check for contamination, each round of PCR included a negative control (without genomic DNA), and there were no products in all negative controls. The PCR products were electrophoresed on 1.5% agarose gels staining with ethidium bromide, and were visualized by ultraviolet transillumination. The purification and sequencing of the PCR products were same with those described in Sun *et al.* (2017).

### Sequence assembly and gene annotation

Sequence assembly and annotation were performed with DNASTAR package (Lasergene version 5.0; Madison, WI, USA). The boundaries of rRNA genes and protein coding genes (PCGs) were detected by aligning our sequences with other available Coraciiform mitogenomes in GenBank: *Ceryle rudis* (NC_024280), *Halcyon pileata* (NC_024198) (Sun *et al.*, 2017), *Halcyon coromanda* (NC_028177) (Park *et al*., unpublished data), *Todirhamphus sanctus* (NC_011712) (Pratt *et al.*, 2009), *Aceros waldeni* (NC_015085) (Sammler *et al.*, 2011), *Bycanistes brevis* (NC_015201) (Pacheco *et al.*, 2011), *Penelopides panini* (HQ834451) (Sammler *et al.*, 2011), *Eurystomus orientalis* (NC_011716) (Pratt *et al.*, 2009), *Merops viridis* (NC_034642) (Huang *et al.*, 2017) and *Upupa epops* (NC_028178) (Park *et al*., unpublished). Gene annotations were conducted with the MITOS webserver (Bernt *et al.*, 2013) and tRNAscan-SE 2.0 (http://lowelab.ucsc.edu/tRNAscan-SE/) (Lowe *et al.*, 2016). Cloverleaf secondary structure and anticodons of tRNA genes were determined with the web-server of the tRNAscan-SE v 2.0 (Lowe *et al.*, 2016). The formulas AT skew = [A-T]/[A+ T] and GC skew = [G-C]/[G + C] were used to calculate the skewness values (Junqueiraa *et al.*, 2004). The relative synonymous codon usage (RSCU) values were measured by MEGA X 10.1 (64-bit) BETA (for Windows) program (Kumar *et al.*, 2018).

### Phylogenetic relationships inferred from mitogenome data

To investigate the phylogenetic relationships among Coraciiformes and other related orders that have been referred in previous studies (de los Monteros, 2000; Hackett *et al.*, 2008; Pacheco *et al.*, 2011; Jetz *et al.*, 2012; Jarvis *et al.*, 2014; Mahmood *et al.*, 2014, Reddy *et al.*, 2017), phylogenetic trees including 30 species belonging to six orders (12 families) were reconstructed with mitogenome sequences. *Gallus gallus* (NC_001323) was chosen as outgroup. The mitogenome information of all species involved were shown in Table S1.

Two different sets of data were used in phylogenetic analyses. In the first set, the control regions (CRs) of all mitogenomes were deleted, because the great differences in CRs would lead to stochastic errors or the effect of homoplasy. The second set of data included the sequences of 12 protein coding genes (PCGs). MT-ND6 was excluded because of the very different evolutionary tendency from other 12 PCGs based on the dN/dS ratios.

MrBayes 3.2.7a (Ronquist and Huelsenbeck, 2003) was applied to construct the Bayesian tree. The program Modeltest version 3.7 (Posada and Crandall, 1998) chose the GTR + I + G model and the GTR+G model as the appropriate substitution model of sequence evolution for the first and second set of data, respectively. The detailed processes for Bayesian tree construction were the same as those reported previously (Sun *et al.*, 2017). We used two independent runs to confirm the convergence of the Bayesian posterior probabilities (BPP) distribution.

## Results and Discussion

### Mitogenome organization and nucleotide composition

The mitogenomes of *A. atthis*, *H. smyrnrnsis* and *M. lugubris* are circular and double-stranded macromolecules, with the size of 17,383 bp, 17,892 bp and 17,223 bp, respectively (Table S2, S3). The accession numbers of the three mitogenomes in GenBank are NC_035868 (KY964271, *A. atthis*), NC_035746 (KY940559, *H. smyrnensis*) and NC_035658 (KY940558, *M. lugubris*). Among 13 available Coraciiform mitogenomes (Table 1), the smallest one is 16,542 bp (*H. coromanda*), and the largest one is 22,737 bp (*P. panini*). Great variation in lengths of control regions (CRs) is the main reason for size difference of these mitogenomes (Table S3).

**Table 1:** Coraciiform species of mitogenomes analyzed in this study.

Family	Species	Accession	Genome Size (bp)	A+T%	AT-skew	GC-skew	Reference
Alcedinidae	*Alcedo atthis*	NC_035868	17,383	55.3	0.085	-0.383	This study
	*Ceryle rudis*	NC_024280	17,355	55.9	0.148	-0.400	Sun *et al.* (2017)
	*Halcyon pileata*	NC_024198	17,612	53.7	0.140	-0.401	Sun *et al.* (2017)
	*Halcyon smyrnensis*	NC_035746	17,892	54.3	0.140	-0.409	This study
	*Halcyon coromanda*	NC_028177	16,542	53.9	0.141	-0.406	DS
	*Megaceryle lugubris*	NC_035658	17,223	55.1	0.143	-0.384	This study
	*Todiramphus sanctus*	NC_011712	17,549	55.2	0.132	-0.394	Pratt *et al.* (2009)
Bucerotidae	*Aceros waldeni*	NC_015085	21,657	55.0	0.133	-0.383	Sammler *et al.* (2011)
	*Bycanistes brevis*	NC_015201	17,591	53.0	0.137	-0.377	Pacheco *et al.* (2011)
	*Penelopides panini*	NC_015087	22,737	55.2	0.136	-0.396	Sammler *et al.* (2011)
Coraciidae	*Eurystomus orientalis*	NC_011716	17,210	53.5	0.127	-0.400	Pratt *et al.* (2009)
Meropidae	*Merops viridis*	NC_034642	18,295	51.9	0.101	-0.400	Huang *et al.* (2017)
Upupidae	*Upupa epops*	NC_028178	16,562	55.4	0.136	-0.371	DS

**Figure 1:**
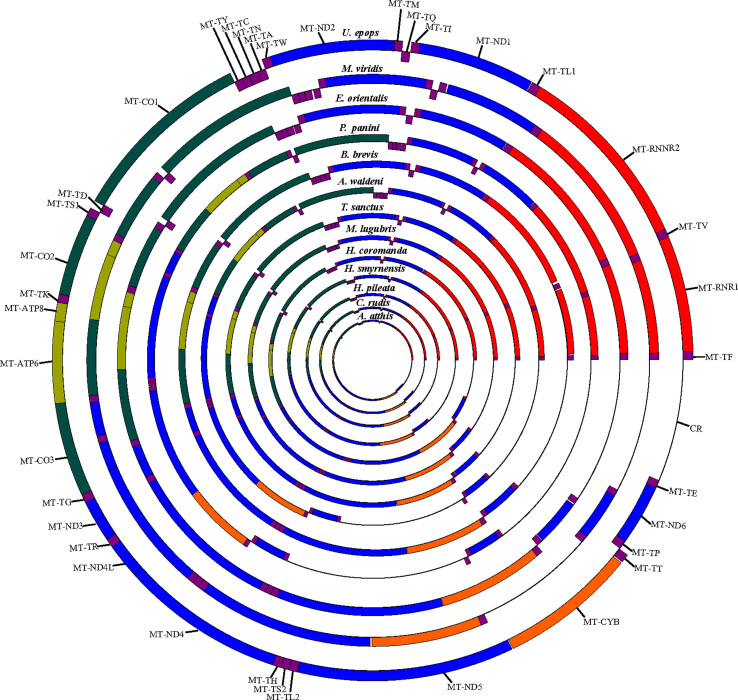
Comparative circular map showing the genome organizations of 13 Coraciiform mitogenomes listed in Table 1.

The mitogenomes of *A. atthis*, *H. smyrnrnsis* and *M. lugubris* contain 37 genes (including 22 tRNA genes, 13 PCGs and two rRNA genes) and one CR (Table S2). Twenty-eight genes (14 tRNA genes, 12 PCGs and two rRNA genes) are located on the H strand, and the remaining nine genes are located on the L strand (Table S2). All genes are compactly arranged, and gene overlaps exist at several gene junctions (Table S2). Gene arrangement of the three mitogenomes is identical to the putative ancestral gene order in avian mitogenomes (Gibb *et al.*, 2007; Song *et al.*, 2015). The comparative circular map is presented to visualize the genome organizations of 13 Coraciiform mitogenomes (Figure 1).

All 13 Coraciiform mitogenomes show nucleotide bias toward A+T in both strands, and display slight positive AT-skews and negative GC-skews (Table 1, Table S3), which suggest that the content of adenine is higher than that of thymine, and the content of cytosine is higher than that of guanine. Except the MT-RNR1 in five species (*A. waldeni*, *B. brevis*, *P. panini*, *M. viridis*, *U. epop*), other fragments in 13 mitogenomes present nucleotide bias toward A and T. The A + T bias in CRs are more significant (Table S3).

### Structure of control regions

CRs of different species are highly variable and distinctive. The length of CR in hte mitogenomes of *A. atthis*, *H. smyrnensis*, and *M. lugubris* is 1,850 bp, 2,333 bp and 1,672 bp, respectively. Among 13 Coraciiform mitogenomes, the longest CR is 5,863 bp in size (*P. panini*) (Table S3).

In addition to great variation in length, complex rearrangements have happened around CRs and flanking genes in some species. To date, at least seven gene orders have been identified in avian mitogenomes: (1) ancestral avian CR; (2) duplicate CR; (3) duplicate TT-CR; (4) duplicate TT-TP and CR; (5) duplicate TE-CR; (6) remnant CR; (7) erroneous gene order (Desjardins and Morais, 1990; Mindell and Sorenson, 1998; Eberhard and Wright, 2001; Abbott *et al.*, 2005; Gibb *et al.*, 2007; Singh *et al.*, 2008; Verkuil *et al.*, 2010; Zhou *et al.*, 2014; Kang *et al.*, 2018). Three types of gene orders were identified in 13 Coraciiform mitogenomes (Figure 1), with ancestral CR type for 10 mitogenomes, duplicate TT-CR for two mitogenomes (*A. waldeni* and *P. panini*), and duplicate CR for one mitogenome (*M. viridis*).

Based on conserved motifs (Sbisà *et al.*, 1997; Pratt and Gibb, 2009; Sammler *et al.*, 2011; Park *et al.*, 2015; Huang *et al.*, 2017; Sun *et al.*, 2017), the CRs of *A. atthis*, *H. smyrnensis*, and *M. lugubris* were divided into three different domains: the peripheral and highly variable domains (I and III), and the conserved domain II (Figure S1). In Domain I, there are two extended blocks (ETAS1-2) including sequences that are responsible for the termination of replication (TAS, 5’-TATAT-3’ and 5’-TACAT-3’) (Figure S1, Table S4). A CSBI-LIKE block (sequence that is similar to the conserved block) also exists in Domain I. There are seven conserved blocks (C, D, E, F, CSBa, b and B) in Domain II of *H. smyrnensis* and *M. Lugubris*, while B-box is absent in Domain II of *A. atthis* (Figure S1, Table S4). Domain III includes a conserved block (CBS1) that is responsible for the regulation of mtDNA replication, a heavy strand replication origin (O_H_), bi-directional transcription promoter of L- and H- strand (LSP/HSP), and a poly (T) sequence at downstream of the CSB1 (Figure S1, Table S4). Besides, there are tandem repeats near the 3’ terminal of Domain III. There are two types of repeats in *A. atthis* (5’-TTCGTTTG-3’ ; 5’-ACAAAACAAACGAATCAATTAGACTTTATCTAC-3’) and *M. lugubris* (5’-CAATTAACGAA-3’; 5’-CATTAACGAA-3’), and three types of repeats in *H. smyrnensis* (5’-AATTCGTTGATC-3’; 5’-TCGTTGATCGAT-3’; 5’-CATAAATTCTGACAAATTAACGAATGAACTCTAATTACACAAGCAGACATTCCCAACAAACAAAAT-3’). The difference in repeat sequences lead to different size of CRs in three species. The origination and evolutionary history of different types of repeat sequences in CRs of animal mitogenomes have not been fully solved (Levinson *et al.*, 1987; Piganeau *et al.*, 2004; Mjelle *et al.*, 2008; Leclercq *et al.*, 2010; Sammler *et al.*, 2011; Shi *et al.*, 2013).

### Structure of transfer and ribosomal RNA genes

All 22 tRNA genes that are common in vertebrate mitogenomes are found in the mitogenomes of *A. atthis*, *H. smyrnensis*, and *M. lugubris* (Figure 1). The lengths of these genes in three mitogenomes are similar (Table S2). All 22 tRNAs can fold into normal clover-leaf secondary structure (Figure S2). Apparent length difference and nucleotide variations exist in the TΨC and DHU loops and stems, while the anticodon loop, anticodon stem, and acceptor stem are more conserved in length. Atypical pairings of G-U and unmatched base pairs of A-C, A-A, U-U, and U-C are scattered throughout the stems (Figure S2). These kinds of mismatched pairs or unmatched base pairs have also been found in other vertebrate and invertebrate mitogenomes (Harlid *et al.*, 1998; Slack *et al.*, 2003; Li *et al.*, 2007; Bi *et al.*, 2012; Zou *et al.*, 2015; Sun *et al.*, 2017; Bi *et al.*, 2019; He *et al.*, 2019).

Two rRNA genes locate between MT-L1 and MT-TF, and they are separated by MT-TV (Figure 1, Table S2). The lengths of the MT-RNR1 in 13 Coraciiform mitogenomes range from 966 bp (*M. lugubris*) to 981 bp (*H. coromanda*) (Table S3). The secondary structures of MT-RNR1 for *A. atthis*, *H. Smyrnensis* and *M. lugubris* are similar, comprising three main domains and 46 helices (Figure S3). Nucleotide substitutions are mainly located at H609, H768, H814 and loops near H307, H655, H683, and H884. The sizes of the MT-RNR2 in 13 Coraciiform mitogenomes range from 1,578 bp (*U. epops*) to 1,614 bp (*M. viridis*) (Table S3). The secondary structures of MT-RNR2 for *A. atthis*, *H. Smyrnensis* and *M. lugubris* contain six domains and 59 helices (Figure S4). The sequences for Domain IV are conserved, while there are many nucleotide substitutions in Domain I-III, V and VI. The secondary structures of two rRNAs in many avian species were similar, while there were obvious differences in secondary structures of mitochondrial rRNAs between birds and bees (De Los Monteros, 2003; Li *et al.*, 2015; Bi *et al.*, 2019; He *et al.*, 2019; this study).

### Characteristics of Protein Coding Genes (PCGs)

All 13 PCGs that are typical in animal mitogenomes are identified in the mitogenomes of *A. atthis*, *H. smyrnensis* and *M. lugubris*, among which only MT-ND6 is encoded by the L strand (Figure 1, Table S2). The lengths of each PCGs in the three mitogenomes are almost the same (Table S5). MT-ND5 (1,815 bp) is the longest one, and MT-ATP8 (168 bp) is the shortest one. Twelve PCGs show nucleotide bias toward A+T, while the MT-ND6 displays slight nucleotide bias toward G+C. Except the MT-ND1, MT-ND3, and MT-CYB of *A. atthis*, other PCGs show a positive AT skews and a great negative GC skew (Table S5). The AT skews for MT-ND6 are significantly greater than those for other PCGs.

ATN is the start codon of most PCGs in three Coraciiform mitogenomes, while the start codon of MT-COI is GTG, and the start codon for MT-ND3 of *A. atthis* is TAA (Table S2). Ten PCGs terminate with AGG, TAA, or TAG, and the remaining three PCGs (MT-CO3, MT-ND2, and MT-ND4) have incomplete stop codons (T), which can be adjusted to a TAA terminal codon by posttranscriptional polyadenylation (Ojala *et al.*, 1981).

The nucleotide composition of MT-ND6 is very different from those of other PCGs, so it is not included in the codon usage analyses. Except the stop codons, 12 PCGs of the three Coraciiform mitogenomes contain 3,796 (*A. atthis*) and 3,793 (*H. smyrnensis* and *M. lugubris*) codons, respectively. The content of A+T in all three codon positions are larger than or equal to 50%. For other 10 Coraciiform mitogenomes, the content of A+T in the second codon positions are greater than 50%, whereas the nucleotide bias toward A+T or G+C in the first and third codon positions are varied in different species (Table S6). The G content in the second and third codons are low, especially, the G content in the third codons are only 3.6% – 6.0% in 13 Coraciiform mitogenomes. The low G content in third codon positions of mitochondrial PCGs is a common feature in mammalian and avian mitogenomes (Harlid *et al.*, 1998; Gibson *et al.*, 2005; Zou *et al.*, 2015; Gong *et al.*, 2017; Sun *et al.*, 2017; Bi *et al.*, 2019). The variation ranges of nucleotide content in three codon positions are negatively correlated to the selective pressure that they are subjected to (Zhong *et al.*, 2002). The second codon positions subject to the greatest selective pressure, corresponding to the smallest nucleotide variation range (Table S6).

Relative synonymous codon frequencies (RSCU) was applied to calculate the codon usage of 12 PCGs of the three Coraciiform mitogenomes, and the results are showed in Figure S5. Corresponding to the high content of A and C in the third codon positions (Table S6), the frequently used codons are NNA and NNC (Figure S5). Eight types of amino acids (L, V, S, P, T, A, R, and G) are frequently used, among which the leucine is the most frequently used amino acids.

The extra insertion of “C” in MT- ND3 (position 174) that was identified in some avian mitogenomes (Harlid *et al.*, 1998; Kan *et al.*, 2010a, 2010b; Yang *et al.*, 2010; Zhang *et al.*, 2012; Ren *et al.*, 2014; Sun *et al.*, 2017; Bi *et al.*, 2019) also exists in nine Coraciiform mitogenomes, including *A. atthis*, *H. smyrnensis* and *M. lugubris* (Figure S6).

### The evolutionary patterns of mitochondrial PCGs among 13 Coraciiform species

The total length of aligned 13 mitochondrial PCGs from 13 Coraciiform species is 11,402 bp (without gaps), and there are 5,483 variable sites (Table 2). Based on the percent of variable sites, the most variable gene is MT-ATP8, followed by MT-ND6, MT-ND2, and MT-ND5. In contrary, the most conserved gene is MT-CO1, then MT-CO2 and MT-CO3 (Table 2). The nucleotide diversity (π) of 13 PCGs varies from 0.145 (MT-CO1) to 0.233 (MT-ATP8). The π value is positively correlated with the percent of variable sites. The ts/tv ratios of 13 PCGs range from 1.21 (MT-ND4L) to 2.72 (MT-ND6), and the dN/dS ratios vary from 0.047 (MT-CO1) to 1.151 (MT-ND6) (Table 2). There was no correlation between the ts/tv and dN/dS ratios.

**Table 2:** Rates and patterns of evolution among mitochondrial PCGs and 13 species of Coraciiformes.

Gene	Length (bp)	Var. sites (%)	π	dN	dS	dN/dS	ts/tv
MT-ATP6	684	323 (47.22%)	0.180	0.061	0.480	0.128	1.27
MT-ATP8	168	100 (59.52%)	0.233	0.153	0.483	0.317	1.24
MT-CO1	1551	565 (36.43%)	0.145	0.023	0.488	0.047	1.72
MT-CO2	684	275 (40.20%)	0.154	0.045	0.480	0.094	1.57
MT-CO3	784	324 (41.33%)	0.151	0.047	0.460	0.103	1.62
MT-CYB	1143	495 (43.31%)	0.172	0.062	0.479	0.129	1.33
MT-ND1	978	476 (48.67%)	0.194	0.066	0.531	0.125	1.64
MT-ND2	1041	599 (57.54%)	0.223	0.120	0.504	0.238	1.55
MT-ND3	352	165 (46.88%)	0.188	0.157	0.281	0.558	1.46
MT-ND4	1378	717 (52.03%)	0.202	0.099	0.473	0.209	1.34
MT-ND4L	297	152 (51.18%)	0.192	0.086	0.479	0.179	1.21
MT-ND5	1820	984 (54.07%)	0.209	0.112	0.478	0.235	1.35
MT-ND6	522	308 (59.00%)	0.217	0.268	0.232	1.151	2.72
overall	11402	5483 (48.09%)	0.189				

Combining our results and previously published data (Liang *et al.*, 2015; Bi *et al.*, 2019), we could find some concordant features on the evolution of avian mitochondrial PCGs: 1) MT-ATP8 is the most diverse gene, while MT-CO1, MT-CO2 and MT-CO3 are very conserved; 2) most PCGs were subjected to negative selection, while the protein evolution rates of these genes vary greatly in different groups; 3) more nucleotide transitions than transversions have happened in all PCGs; 4) in different groups, the evolutionary patterns of the MT-ND6 are different. The dN/dS ratio of the MT-ND6 among Coraciiform species is greater than 1 (Table 2), indicating positive selection or relaxed purifying selection effect.

### Phylogenetic relationships among 30 species based on mitogenome data

The relationships among Coraciiformes and several related orders were in debate both in morphological taxonomic studies (Cracraft, 1981, 1988; Burton, 1984; Höfling and Alvarenga, 2001; Mayr, 2003, 2005) and molecular phylogenetic studies using nuclear DNA (Hackett *et al.*, 2008; Jetz *et al.*, 2012; Jarvis *et al.*, 2014; Reddy *et al.*, 2017) and mtDNA (de los Monteros, 2000; Pacheco *et al.*, 2011; Mahmood *et al.*, 2014;). Biological factors such as incomplete lineage sorting, hybridization and adaptive introgression, demographic disparities and sex-biased asymmetries (Toews and Brelsford, 2012) and potential systematic errors in analysis methods (Brinkmann and Philippe, 2008) often lead to nuclear and mitochondrial phylogenetic discordance in many animal systems. Analyses with single genes would increase stochastic errors and the effect of homoplasy (Campbell and Lapointe 2011). Reconstructing phylogenetic trees with both mitochondrial and nuclear genome sequences is a new strategy to solve evolutionary puzzles in animals. Here, the mitogenome sequences of 30 species in 12 families are used to explore the phylogenetic placements of Coraciiformes and other five orders. *G. gallus* (NC_001323) is used as outgroup (Table S1).

**Figure 2:**
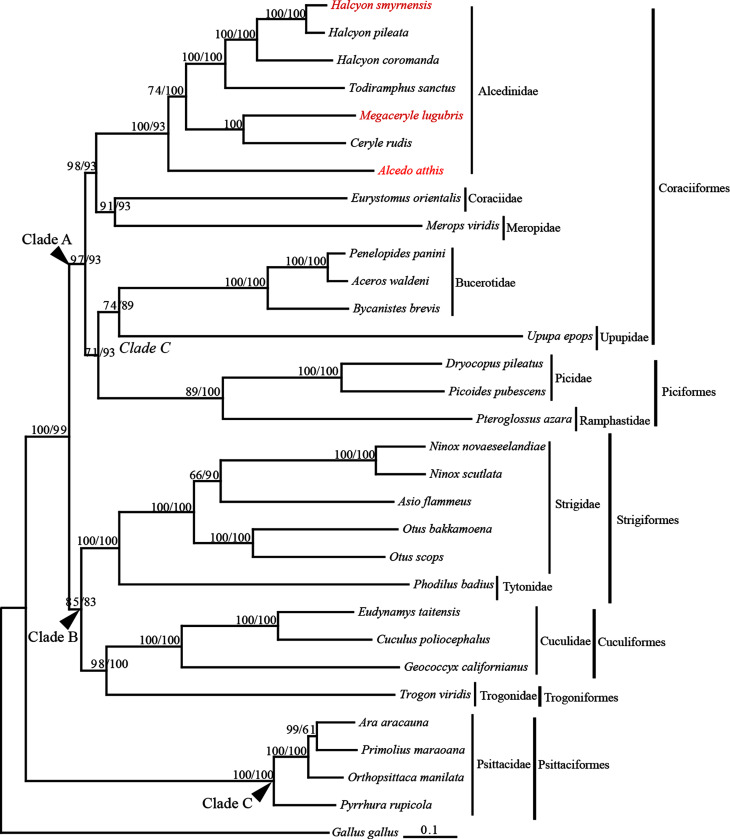
Phylogenetic tree of 30 species from 12 families of six orders with *G. gallus* as outgroups. Analyses were based on mitogenome sequences without control regions and the sequences of 12 PCGs without MT-ND6. The Bayesian posterior probability (PP) values for each node were presented. Values for the first set of data were listed in front of those for the second set of data.

The phylogenetic trees inferred from two sets of data have the same topologies (Figure 2). The topologies are also consistent with the results based on mitogenome data reported by Sun *et al.* (2017) though four new species were added in the present analyses. The monophyly of all 12 families are well supported, and they cluster into three distinct clades (Figure 2). Clade A consists of two subclades: one subclade contains species of Alcedinidae, Coraciidae and Meropidae; another subclade contains species of Bucerotidae, Upupidae, and Picidae. Clade B also comprises two subclades: one subclade includes species of Strigidae and Tytonidae; another subclade includes species of Cuculidae and Trogonidae. Clade A and clade B are sister groups. Clade C contains species from Psittacidae, which is the basal clade of the tree.

The relationships among three families of Coraciiformes (Alcedinidae, Coraciidae and Meropidae) displayed in our trees (Figure 2) are congruous with the results of newly published phylogenetic research based on mitochondrial DNA markers (Tamashiro *et al.*, 2019). Our tree clearly shows that Bucerotidae and Upupidae are more closely related to Picidae (Piciformes) than to other families of Coraciiformes (Figure 2). Our results support a previous proposal that Bucerotidae and Upupidae should be separated from Coraciiformes, which was inferred from phylogenetic studies using morphological characters (Burton, 1984; Olson, 1985; Mayr, 2003;), nuclear DNA (e.g. Sibley and Ahlquist, 1990; Johansson *et al.*, 2001; Hackett *et al.*, 2008; Jetz *et al.*, 2012; Ödeen and Hästad, 2013; Jarvis *et al.*, 2014; Prum *et al.*, 2015; Reddy *et al.*, 2017) and mtDNA (e.g. de los Monteros, 2000; Ericson *et al.*, 2006; Pacheco *et al.*, 2011; Mahmood *et al.*, 2014; Sun *et al.*, 2017; Tamashiro *et al.*, 2019). Our tree also displays that Coraciiformes is more closely related to Picidae (Piciformes) than to Trogonidae (Trogoniformes), which agreed with phylogenetic investigations based on both nuclear DNA (Hackett *et al.*, 2008; Jetz *et al.*, 2012; Ödeen and Hästad, 2013; Jarvis *et al.*, 2014) and mtDNA (Ericson *et al.*, 2006; Tamashiro *et al.*, 2019). However, the relationships among classical Coraciiformes (Alcedinidae, Coraciidae and Meropidae), “Bucerotes” (Bucerotidae and Upupidae) and Picidae (Piciformes) presented in our tree are inconsistent with the results of these studies.

Previous investigations involving different taxa and gene markers (nuclear DNA: Johansson *et al.*, 2001; Hackett *et al.*, 2008; Jarvis *et al.*, 2014; mitochondrial DNA: de los Monteros, 2000; Ericson *et al.*, 2006; Pacheco *et al.*, 2011) did not obtain accordant conclusions on the phylogenetic placement of Trogoniformes. Our results show that, among six orders involved in this study, Trogonidae (Trogoniformes) and Cuculidae (Cuculiformes) are the closest relatives, and that they form a sister taxon to Strigidae (Strigiformes) (Figure 2). In our tree, Psittacidae (Psittaciformes) is the basal clade, indicating distant relationships between Psittaciformes and other five orders (Figure 2). The result is congruous with the conclusion of studies using nuclear (Jetz *et al.*, 2012; Jarvis *et al.*, 2014) and mitochondrial DNA markers (Ericson *et al.*, 2006; Pratt *et al.*, 2009; Tamashiro *et al.*, 2019).

The limitation on taxa in this study would be one of the reasons for inconsistent results between our study and previous researches. More Coraciiform mitogenome data are necessary to resolve the phylogenesis of Coraciiformes and to test the results in previous studies. The new mitogenome data presented in this paper represent a contribution to this long-term goal.

## Supplementary material

The following online material is available for this article:

Table S1 - Species involved in phylogeny analyses of this study.

Table S2 - Organization of mitogenomes of the *A. atthis*, *H. smyrnensis* and *M. lugubris*

Table S3 - Genomic characteristics of 13 coraciiform mtDNA

Table S4 - Sequences of conserved motifs in control regions of *A. atthis*, *H. smyrnensis* and *M. lugubris*

Table S5 - Lengths and base compositions for protein coding genes in mitogenomes of *A. atthis*/*H. smyrnensis*/*M. lugubris*

Table S6 - Nucleotide compositions in three codon positions of 12 mitochondrial protein coding genes.

Figure S1 - Structure diagram of control region (CR) in mitogenomes of *A. atthis*, *H. smyrnensis* and *M. lugubris*.

Figure S2 - Secondary structures of the 22 tRNAs of three species.

Figure S3 - Predicted secondary structures of MT-RNR1 in *A. atthis*.

Figure S4 - Predicted secondary structures of MT-RNR2 in *A. atthis*.

Figure S5 - Relative synonymous codon usage (RSCU) in the mitogenome of *A. atthis*, *H. smyrnensis* and *M. lugubris*.

Figure S6 - Alignment of the mitochondrial MT-ND3 gene region spanning the extra base site (designated by *) for 13 species.
