# Supplementary material for: Characterization of three new mitochondrial genomes of Coraciiformes (Megaceryle lugubris, Alcedo atthis, Halcyon smyrnensis) and insights into their phylogenetics
Source: Genet Mol Biol. 2020 Oct 5;43(4):e20190392. doi: 10.1590/1678-4685-GMB-2019-0392 (PMC7539371; doi:10.1590/1678-4685-GMB-2019-0392)
Supplement: Supplementary file 1 [file 1415-4757-GMB-43-4-e20190392-suppl1.pdf]

Genetics and Molecular Biology
https://doi.org/10.1590/1678-4685-GMB-2019-0392

Supplementary Material to "Characterization of three new mitochondrial genomes of Coraciiformes (*Megaceryle lugubris*, *Alcedo atthis*, *Halcyon smyrnensis*) and insights into their phylogenetics"

Table S1 - Species involved in phylogeny analyses of this study.

Order	Family	Species	Accession	Reference
**Coraciiformes**	Alcedinidae	*Alcedo atthis*	NC_035868	This study
		Ceryle rudis	NC_024280	Sun *et al.* (2017)
		Halcyon pileata	NC_024198	Sun *et al.* (2017)
		Halcyon smyrnensis	NC_035746	This study
		Halcyon coromanda	NC_028177	Park *et al.* (2015a)
		Megaceryle lugubris	NC_035658	This study
		Todiramphus sanctus	NC_011712	Pratt *et al.* (2009)
	Bucerotidae	*Aceros waldeni*	NC_015085	Sammler *et al.* (2011)
		Bycanistes brevis	NC_015201	Pacheco *et al.* (2011)
		Penelopides panini	NC_015087	Sammler *et al.* (2011)
	Coraciidae	*Eurystomus orientalis*	NC_011716	Pratt *et al.* (2009)
	Meropidae	*Merops viridis*	NC_034642	Huang *et al.* (2017)
	Upupidae	*Upupa epops*	NC_028178	Park *et al.* (2015b)
**Cuculiformes**	Cuculidae	*Cuculus poliocephalus*	NC_028414	Wang *et al.* (2016)
		Eudynamys taitensis	NC_011709	Pratt *et al.* (2009)
		Geococcyx californianus	NC_011711	Pratt *et al.* (2009)
**Piciformes**	Picidae	*Dryocopus pileatus*	NC_008546	Gibb *et al.* (2007)
		Picoides pubescens	NC_027936	Zhang *et al.* (2016)
	Ramphastidae	*Pteroglossus azara*	NC_008549	Gibb *et al.* (2007)
**Psittaciformes**	Psittacidae	*Orthopsittaca manilata*	NC_029161	DS
		Pyrrhura rupicola	NC_028404	DS
		Primolius maraoana	NC_029322	Urantowka and Mackiewicz 2015

Order	Family	Species	Accession	Reference
		Ara aracauna	NC_029319	Urantowka and Mackiewicz 2015
**Strigiformes**	Strigidae	*Asio flammeus*	NC_027606	Zhang *et al.* (2016)
		Ninox scutlata	NC_029384	DS
		Ninox novaeseelandiae	NC_005932	Harrison *et al.* (2004)
		Otus bakkamoena	NC_028163	DS
		Otus scops	NC_028162	DS
	Tytonidae	*Phodilus badius*	NC_023787	Mahmood *et al.* (2014)
**Trogoniformes**	Trogonidae	*Trogon viridis*	NC_011714	Pratt *et al.* (2009)
**Galliformes**	Phasianidae	*Gallus gallus*	NC_001323	Valverde *et al.* (1994)

Reference

Gibb GC, Kardailsky O, Kimball RT, Braun EL and Penny D (2007) Mitochondrial genomes and avian phylogeny: Complex characters and resolvability without explosive radiations. Mol Biol Evol 24:269-280.

Harrison GL, McLenachan PA, Phillips MJ, Slack KE, Cooper A and Penny D (2004) Four new avian mitochondrial genomes help get to basic evolutionary questions in the late cretaceous. Mol Biol Evol 21:974-983.

Mahmood MT, McLenachan PA, Gibb GC and Penny D (2014) Phylogenetic position of avian nocturnal and diurnal raptors. Genome Biol Evol 6:326-332.

Pacheco MA, Battistuzzi FU, Lentino M, Aguilar RF, Kumar S and Escalante AA (2011) Evolution of modern birds revealed by mitogenomics: Timing the radiation and origin of major orders. Mol Biol Evol 28:1927-1942.

Pratt RC, Gibb GC, Morgan-Richards M, Phillips MJ, Hendy MD and Penny D (2009) Toward resolving deep neoaves phylogeny: Data, signal enhancement, and priors. Mol Biol Evol 26:313-326.

Sammler S, Bleidorn C and Tiedemann R (2011) Full mitochondrial genome sequences of two endemic Philippine hornbill species 0(Aves: Bucerotidae) provide evidence for pervasive mitochondrial DNA recombination. BMC Genomics 12:35.

Valverde JR, Marco R and Garesse R (1994) A conserved heptamer motif for ribosomal RNA transcription termination in animal mitochondria. Proc Natl Acad Sci USA 91:5368-5371.

Wang N, Liang B, Huo J and Liang W (2016) Complete mitochondrial genome and the phylogenetic position of the Lesser Cuckoo, *Cuculus poliocephalus* (Aves: Cuculiformes). Mitochondrial DNA 27:4409-4410.

Zhang Z, An M, Deng Y and Zhu S (2016) The complete mitochondrial genome of the Downy woodpecker, *Picoides pubescens* (Piciformes: Picidae). Mitochondrial DNA 27:3479-3480.

Zhang Y, Song T, Pan T, Sun X, Sun Z, Qian L and Zhang B (2016) Complete sequence and gene organization of the mitochondrial genome of *Asio flammeus* (Strigiformes, strigidae). Mitochondrial DNA 27:2665-2667.

Huang ZH, Tu FY and Ke DH (2017) Complete Mitochondrial Genome of Blue-Throated Bee-eater *Merops viridis* (Coraciiformes: Meropidae) with its Taxonomic Consideration. Pakistan Journal of Zoology 49:79-84.

Sun X, Zhao R, Zhang T, Gong J, Jing M and Huang L (2017). Two mitochondrial genomes in Alcedinidae (*Ceryle rudis/Halcyon pileata*)

and the phylogenetic placement of Coraciiformes. Genetica 145:431-440.

Urantowka AD and Mackiewicz P (2015) Complete mitochondrial genome of Blue-winged Macaw (*Primolius maracana*). Mitochondrial DNA. Mitogenome Announcement 28:275-276.

Internet Resources

Park CE, Park GS, Jung BK, Park YJ, Kim MC, Park HC and Shin JH (2015a) Halcyon coromanda mitochondrion, complete genoma, http://www.ncbi.nlm.nih.gov/nuccore/NC_028177.

Park CE, Park GS, Jung BK, Park YJ, Kim MC, Park HC and Shin JH (2015b) Upupa epops mitochondrion, complete genome, http://www.ncbi.nlm. nih.gov/nuccore/NC_028178.
