# Supplementary material for: Characterization of three new mitochondrial genomes of Coraciiformes (Megaceryle lugubris, Alcedo atthis, Halcyon smyrnensis) and insights into their phylogenetics
Source: Genet Mol Biol. 2020 Oct 5;43(4):e20190392. doi: 10.1590/1678-4685-GMB-2019-0392 (PMC7539371; doi:10.1590/1678-4685-GMB-2019-0392)
Supplement: Supplementary file 2 [file 1415-4757-GMB-43-4-e20190392-suppl2.pdf]

Genetics and Molecular Biology
https://doi.org/10.1590/1678-4685-GMB-2019-0392

Supplementary Material to "Characterization of three new mitochondrial genomes of Coraciiformes (*Megaceryle lugubris*, *Alcedo atthis*, *Halcyon smyrnensis*) and insights into their phylogenetics"

Table S2 - Organization of mitogenomes of the *A. atthis*, *H. smyrnensis* and *M. lugubris*

Gene	Position[a]		Size(bp)	Spacer(+)/ Overlap(-)	Strand[b]	Codon	
	From	To				Start[c]	Stop[c]
MT-TF	1/1/1	64/69/67	64/69/67	0/0/0	H		
MT-RNR1	65/70/68	1038/1042/1033	974/973/966	0/0/0	H		
MT-TV	1039/1043/1034	1109/1112/1103	71/70/70	0/0/0	H		
MT-RNR2	1110/1113/1104	2697/2698/2692	1588/1586/1589	0/0/0	H		
MT-TL1	2698/2699/2693	2771/2772/2766	74/74/74	9/24/16	H		
MT-ND1	2781/2797/2783	3758/3774/3760	978/978/978	-2/-2/-3	H	ATG/ATG/ATG	AGG/AGG/AGG
MT-TI	3757/3772/3758	3828/3846/3831	72/73/74	8/8/6	H		
MT-TQ	3837/3855/3838	3907/3925/3908	71/71/71	0/0/-1	L		
MT-TM	3908/3926/3908	3975/3993/3976	68/68/69	0/0/0	H		
MT-ND2	3976/3994/3977	5014/5029/5012	1039/1036/1036	0/0/0	H	ATG/ATA/ATA	T[d]/T[d]/T[d]
MT-TW	5015/5030/5013	5086/5105/5087	72/76/75	1/1/1	H		
MT-TA	5088/5107/5089	5156/5175/5157	69/69/69	2/8/14	L		
MT-TN	5169/5184/5172	5242/5256/5245	74/73/74	2/2/2	L		
MT-TC	5245/5259/5248	5311/5326/5314	67/68/67	0/-1/-1	L		
MT-TY	5311/5326/5314	5381/5396/5384	71/71/71	1/1/1	L		
MT-CO1	5383/5398/5386	6933/6948/6936	1551/1551/1551	-9/-9/-9	H	GTG/GTG/GTG	AGG/AGG/AGG
MT-TS1	6925/6940/6928	6998/7012/7001	74/74/74	3/2/2	L		
MT-TD	7002/7015/7004	7070/7085/7074	69/69/71	1/1/1	H		
MT-CO2	7072/7087/7076	7755/7770/7759	684/684/684	2/0/0	H	ATG/ATG/ATG	TAA/TAA/TAA
MT-TK	7758/7771/7760	7827/7842/7832	70/70/73	2/1/1	H		
MT-ATP8	7830/7844/7834	7997/8011/8001	168/168/168	-10/-10/-10	H	ATG/ATG/ATG	TAA/TAA/TAA
MT-ATP6	7988/8002/7992	8671/8685/8675	684/684/684	-1/-1/-1	H	ATG/ATG/ATG	TAA/TAA/TAA
MT-CO3	8671/8685/8675	9454/9468/9458	784/784/784	0/0/0	H	ATG/ATG/ATG	T[d]/T[d]/T[d]
MT-TG	9455/9469/9459	9523/9537/9527	69/69/69	0/0/0	H		
MT-ND3	9524/9538/9528	9875/9889/9879	352/352/352	2/4/4	H	TAA/ATT/ATA	TAA/TAG/TAG
MT-TR	9878/9894/9884	9947/9962/9952	70/69/69	1/1/1	H		
MT-ND4L	9949/9964/9954	10245/10260/10250	297/297/297	-7/-7/-7	H	ATG/ATG/ATG	TAA/TAA/TAA
MT-ND4	10239/10254/10244	11616/11631/11620	1378/1378/1377	0/0/1	H	ATG/ATG/ATG	T[d]/T[d]/TAA
MT-TH	11617/11632/11622	11686/11701/11691	70/70/70	0/0/0	H		
MT-TS2	11687/11702/11692	11751/11766/11756	65/65/65	0/0/0	H		
MT-TL2	11752/11767/11757	11823/11838/11827	72/72/71	0/0/0	H		
MT-ND5	11824/11839/11828	13638/13653/13642	1815/1815/1815	10/13/12	H	ATG/ATG/ATG	TAA/TAA/TAA
MT-CYB	13649/13667/13655	14791/14809/14797	1143/1143/1143	1/0/1	H	ATG/ATG/ATG	TAA/TAA/TAA
MT-TT	14793/14810/14799	14861/14878/14867	69/69/69	2/8/7	H		
MT-TP	14864/14887/14	14933/14957/14	70/70/72	8/8/9	L		

Genetics and Molecular Biology
https://doi.org/10.1590/1678-4685-GMB-2019-0392

Gene	Position[a]		Size(bp)	Spacer(+)/ Overlap(-)	Strand[b]	Codon	
	From	To				Start[c]	Stop[c]
	875	946					
MT-ND6	14942/14965/14956	15463/15486/15477	522/522/522	16/3/3	L	ATG/ATG/ATG	TAA/TAA/TAG
MT-TE	15465/15490/15481	15533/15559/15551	69/70/71	0/0/0	L		
Control region	15534/15560/15552	17383/17892/17223	1580/2333/1672	0/0/0	H		

a Position numbering starts with the 5′ position of control region
b Genes transcripted from the L or H strand
c Start and stop codons of protein coding genes
d Protein coding genes overlapping with tRNA genes end with an incomplete stop codon
