# Supplementary material for: Characterization of three new mitochondrial genomes of Coraciiformes (Megaceryle lugubris, Alcedo atthis, Halcyon smyrnensis) and insights into their phylogenetics
Source: Genet Mol Biol. 2020 Oct 5;43(4):e20190392. doi: 10.1590/1678-4685-GMB-2019-0392 (PMC7539371; doi:10.1590/1678-4685-GMB-2019-0392)
Supplement: Supplementary file 3 [file 1415-4757-GMB-43-4-e20190392-suppl3.pdf]

Genetics and Molecular Biology
https://doi.org/10.1590/1678-4685-GMB-2019-0392

Supplementary Material to "Characterization of three new mitochondrial genomes of Coraciiformes (*Megaceryle lugubris*, *Alcedo atthis*, *Halcyon smyrnensis*) and insights into their phylogenetics"

Table S3 - Genomic characteristics of 13 coraciiform mtDNA.

	Gene on H-strand		Gene on L-strand		MT-RNR2		MT-RNR1		MT-tRNA gene		Control region		mtDNA			
	Length	AT%	Length	AT%	Length	AT%	Length	AT%	Length	AT%	Length	AT%	Length	AT%	AT-skew	GC-skew
Alcedo atthis	16,296	55.7	1087	55.1	1588	55.2	974	51.6	1543	57.8	1850	59.8	17,383	55.3	0.085	-0.383
Ceryle rudis	16,238	55.9	1117	55.3	1585	55.8	971	52.0	1553	57.5	1874	65.5	17,355	55.9	0.148	-0.400
Halcyon pileata	16,517	53.7	1095	51.5	1589	53.7	973	51.0	1552	56.1	2057	61.9	17612	53.7	0.140	-0.401
Halcyon smyrnensis	16804	54.5	1088	54.2	1586	54.2	973	52.3	1550	56.5	2333	62.9	17892	54.3	0.140	-0.409
Halcyon coromanda	15445	53.7	1097	54.8	1585	53.5	981	51.5	1540	57.6	986	57.6	16542	53.7	0.141	-0.406
Megaceryle lugubris	16,138	55.3	1085	55.2	1589	55.7	966	52.2	1557	57.0	1672	64.1	17,223	55.1	0.143	-0.384
Todirhamphus sanctus	16,430	55.3	1119	53.8	1587	54.2	975	51.0	1544	57.2	2088	65.2	17.549	55.3	0.132	-0.394
Aceros waldeni	20,516	55.2	1141	53.1	1601	53.3	973	48.6	1776	56.3	4792	66.5	21,657	55.1	0.133	-0.383
Bycanistes brevis	16,492	53.1	1097	52.4	1597	54.5	978	49.3	1540	56.8	2026	61.4	17,591	53.1	0.137	-0.377
Penelopides panini	21,516	55.4	1131	52.3	1598	53.5	973	48.0	1777	56.3	5863	65.8	22,737	55.2	0.136	-0.396
Eurystomus orientalis	16,064	53.6	1146	52.1	1585	53.5	975	50.9	1548	53.5	1636	62.5	17210	53.4	0.127	-0.400
Merops viridis	17,210	54.9	1085	55.4	1614	53.2	978	48.4	1559	56.7	2711	57.0	18,295	52.0	0.101	-0.400
Upupa epops	15,469	55.6	1093	55.9	1578	54.3	968	49.5	1541	57.8	1052	63.3	16,562	55.4	0.136	-0.371
Average	16,909	54.7	1106	53.9	1591	54.2	974	50.5	1583	56.7	2390	62.6	15,504	54.4		
Range	6071	2.8	61	4.4	36	2.6	15	4.3	236	4.3	4877	9.5	6195	3.9		
