# Supplementary material for: Characterization of three new mitochondrial genomes of Coraciiformes (Megaceryle lugubris, Alcedo atthis, Halcyon smyrnensis) and insights into their phylogenetics
Source: Genet Mol Biol. 2020 Oct 5;43(4):e20190392. doi: 10.1590/1678-4685-GMB-2019-0392 (PMC7539371; doi:10.1590/1678-4685-GMB-2019-0392)
Supplement: Supplementary file 4 [file 1415-4757-GMB-43-4-e20190392-suppl7.pdf]

Genetics and Molecular Biology
https://doi.org/10.1590/1678-4685-GMB-2019-0392

Supplementary Material to "Characterization of three new mitochondrial genomes of Coraciiformes (*Megaceryle lugubris*, *Alcedo atthis*, *Halcyon smyrnensis*) and insights into their phylogenetics"

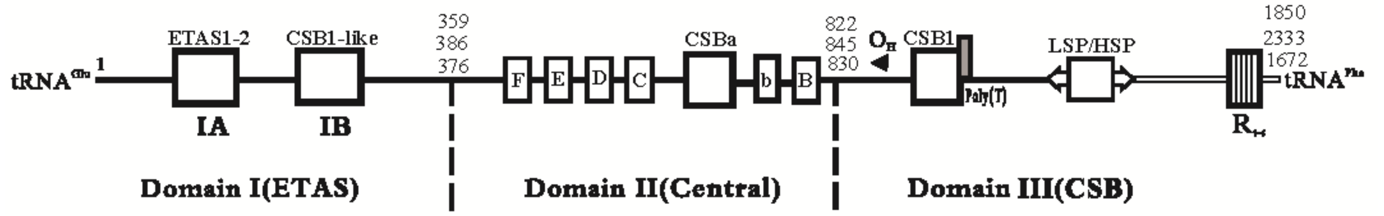

Figure S1 - Structure diagram of control region (CR) in mitogenomes of *A. atthis*, *H. smyrnensis* and *M. lugubris*. Positions of the conserved boxes and the division into the three domains, Domain I (ETAS), Domain II (Central), Domain III (CSB) are shown. ETAS =extended termination-associated sequences; F to B boxes = conserved sequence boxes in the central domain; CSB = conserved sequence block; CSB-like = a sequence similar to the CSB; LSP = light-strand transcription promoter; HSP = heavy-strand transcription promoter. All specific sequences of the conserved boxes in the three mitogenomes were listed in Table S4.
