# Supplementary material for: Characterization of three new mitochondrial genomes of Coraciiformes (Megaceryle lugubris, Alcedo atthis, Halcyon smyrnensis) and insights into their phylogenetics
Source: Genet Mol Biol. 2020 Oct 5;43(4):e20190392. doi: 10.1590/1678-4685-GMB-2019-0392 (PMC7539371; doi:10.1590/1678-4685-GMB-2019-0392)
Supplement: Supplementary file 5 [file 1415-4757-GMB-43-4-e20190392-suppl4.pdf]

Genetics and Molecular Biology
https://doi.org/10.1590/1678-4685-GMB-2019-0392

Supplementary Material to “Characterization of three new mitochondrial genomes of Coraciiformes (*Megaceryle lugubris*, *Alcedo atthis*, *Halcyon smyrnensis*) and insights into their phylogenetics”

Table S4 - Sequences of conserved motifs in control regions of *A. atthis*, *H. smyrnensis* and *M. lugubris.*

Speices	region	position	Sequence（5`-3`）
A. atthis	ETAS1-2	64-205	TTCTGTGCATGGGTTTATTTTCCCCATTACTAATGCATTCTAC TAGGACATTTTATGTATGCACGATAGACATTATACCCAATTTC TTACCCCCATTAATTTTTCATAACCGGGCATATTAAATATCTC CCCATTCCTACC
H. smyrnensis		79-220	TATTTTTATGCCCCCTTATTCTTTGGCCTCAATTAGGACATTA TACTTAATGTACGAATTACATTACATGTAATGGTTTACCCTCA AAAAACTTTTTATACTCGGGCATAAAAATACTGCACAAAGT CATACCTAACTGGACT
M. lugubris		79-216	AACTTATAATCCCCATTTATCATTGCACTCAACTAGGACATT ACAATTCATGTACAATCCACATTACACTTAATGTTTGATCCA CATACAAATATTATACCCGGGCATACATTTATCTAACACATCT CTACATTCAAGC
A. atthis	CSB1-like	323-346	TGCACCGTCACAGGCCATGAATCG
H. smyrnensis		328-352	AATTATGTACGCTCCGGGGTGAAA
M. lugubris		331-354	TGCATGGTCGCAGGTCATATCTCG
A. atthis	F-box	397-421	TGACCCATCACGTGAAATCAGCAACGC
H. smyrnensis		418-443	GGACACTCACGTGAAATCAGCAACGC
M. lugubris		405-443	GGACACTCACGTGAAATCAGCAACGC
A. atthis	E-box	443-464	GTTCCTAGCTTCAGGCCCATTC
H. smyrnensis		463-484	GTCCCTAGCTTCAGGCCCATAC
M. lugubris		451-472	GTCCCTAGCTTCAGGCCCATAC
A. atthis	D-box	502-524	CCTCTGGTTCCTCGGTCAGGACC
H. smyrnensis		523-545	CCTCTGGTTCCTCTGTCAGGGCC
M. lugubris		511-533	CCTCTGGTTCCTCTGTCAGGGCC
A. atthis	C-box	548-574	TTGCCCTTCACAGAGCCATTTGGTTGG
H. smyrnensis		572-597	TCGCCTTTCATGAGGCATTTGGTTGG
M. lugubris		557-582	TCGCCTTTCATGAGGCATTTGGTTGG
A. atthis	CSBa	626-663	TCTCTTCTTTTTTTCTGCGTAGTCTTCAGGTAGCCCT
H. smyrnensis		654-688	GTATTTCTTTTTTTGGGGCGTCTTCAGGTAGCCCT
M. lugubris		633-672	CCTTCCCTTTTTTTCTTTTGGGGCGTCTTCAGGTAGCCCT
A. atthis	b-box	786-800	CGCTGATGCACTTTG
H. smyrnensis		807-821	CACTGATGCACTTTG
M. lugubris		791-805	CACTGATGCACTTTG
A. atthis	B-box	/	/
H. smyrnensis		825-841	TCGCATTTGGTTATGGT
M. lugubris		808-824	GTCCATTTGGTTATGGT
A. atthis	CSB1	842-867	TATTTAGATGAATGCTTGTTGGACATG
H. smyrnensis		869-894	TATTTAGTGAATGCTTGTAGGACATA
M. lugubris		853-878	TATTTAGTGAATGCTTGTAGGACATA
A. atthis	LSP/HSP	877-896	CTTACTTTTCACACGTCAAT
H. smyrnensis		922-948	TTTCTTAGCAGAATCAGTAAATTCCAG
M. lugubris		906-932	TTTTCTAACAACACTAGTAACTTTCAA
