# Supplementary material for: Characterization of three new mitochondrial genomes of Coraciiformes (Megaceryle lugubris, Alcedo atthis, Halcyon smyrnensis) and insights into their phylogenetics
Source: Genet Mol Biol. 2020 Oct 5;43(4):e20190392. doi: 10.1590/1678-4685-GMB-2019-0392 (PMC7539371; doi:10.1590/1678-4685-GMB-2019-0392)
Supplement: Supplementary file 9 [file 1415-4757-GMB-43-4-e20190392-suppl5.pdf]

Genetics and Molecular Biology
https://doi.org/10.1590/1678-4685-GMB-2019-0392

Supplementary Material to "Characterization of three new mitochondrial genomes of Coraciiformes (*Megaceryle lugubris*, *Alcedo atthis*, *Halcyon smyrnensis*) and insights into their phylogenetics"

Table S5 - The lengths and base compositions for protein coding genes in mitogenomes of *A. atthis*/*H. smyrnensis*/ *M. lugubris.*

Gene	Length(bp)	Proportion of nucleotides (%)					AT Skew	GC Skew
		A	C	G	T	A+T		
MT-ND1	978/978/978	26.9/27.4/29.0	33.1/34.6/31.1	12.9/13.2/13.7	27.1/24.8/26.2	54.0/52.2/55.2	-0.004/0.049/0.051	-0.440/-0.448/-0.388
MT-ND2	1039/1036/1036	31.7/32.2/33.1	34.1/34.8/33.5	10.1/9.5/10.6	24.2/23.5/22.8	55.9/55.7/55.9	0.134/0.156/0.184	-0.543/-0.571/-0.518
MT-CO1	1551/1551/1551	27.3/27.5/26.8	32.2/31.7/31.3	15.7/15.8/16.8	24.8/25.0/25.1	52.1/52.5/51.9	0.048/0.047/0.033	-0.332/-0.335/-0.303
MT-CO2	684/684/684	30.1/30.6/31.4	31.6/32.6/31.0	13.9/14.0/14.0	24.4/22.8/23.5	54.5/53.4/54.9	0.105/0.146/0.144	-0.409/-0.399/-0.376
MT-ATP8	168/168/168	36.3/37.5/37.5	32.7/36.9/32.7	6.5/4.8/5.4	24.4/20.8/24.4	60.7/58.3/61.9	0.196/0.286/0.212	-0.667/-0.770/-0.719
MT-ATP6	684/684/684	28.9/28.8/29.4	33.8/37.9/36.1	10.2/9.5/10.1	27.0/23.8/24.4	55.9/52.6/53.8	0.034/0.095/0.093	-0.536/-0.599/-0.563
MT-CO3	784/784/784	28.7/29.1/29.1	28.6/32.9/32.3	14.7/14.8/14.8	28.1/23.2/23.9	56.8/52.3/53.0	0.011/0.113/0.098	-0.321/-0.380/-0.371
MT-ND3	352/352/352	25.0/28.4/27.8	32.4/34.7/34.4	12.5/11.4/11.9	30.1/25.6/25.9	55.1/54.0/53.7	-0.093//0.052/0.035	-0.443/-0.506/-0.485
MT-ND4L	297/297/297	27.6/27.9/30.0	31.6/33.7/34.0	14.1/13.1/12.5	26.6/25.3/23.6	54.2/53.2/53.6	0.018/0.049/0.119	-0.382/-0.439/-0.464
MT-ND4	1377/1378/1377	28.6/30.0/30.3	34.9/37.9/35.8	9.9/10.5/10.7	26.6/21.6/23.2	55,2/51.6/53.5	0.036/0.136/0.162	-0.611/-0.566/-0.540
MT-ND5	1815/1815/1815	30.7/31.7/32.2	34.2/35.9/33.7	10.8/10.8/11.0	24.3/21.5/23.1	55.0/53.2/55.3	0.116/0.192/0.164	-0.520/-0.538/-0.507
MT-CYB	1143/1143/1143	27.1/26.9/28.3	33.5/36.9/35.5	12.2/12.3/12.7	27.2/23.9/23.5	54.3/50.8/51.8	-0.002/0.059/0.093	-0.467/-0.499/-0.474
MT-ND6	522/522/522	37.7/39.1/40.0	41.8/42.9/42.1	9.8/8.2/9.0	10.7/9.8/8.8	48.4/48.9/48.8	0.557/0.599/0.639	-0.620/-0.679/-0.648
Average		29.7/27.5/31.1	33.4/33.7/34.1	11.8/13.3/11.8	25.0/25.5/23.0	54.7/53.0/54.1	0.086/0.048/0.150	-0.478/-0.434/-0.486
