# Supplementary material for: Characterization of three new mitochondrial genomes of Coraciiformes (Megaceryle lugubris, Alcedo atthis, Halcyon smyrnensis) and insights into their phylogenetics
Source: Genet Mol Biol. 2020 Oct 5;43(4):e20190392. doi: 10.1590/1678-4685-GMB-2019-0392 (PMC7539371; doi:10.1590/1678-4685-GMB-2019-0392)
Supplement: Supplementary file 10 [file 1415-4757-GMB-43-4-e20190392-suppl6.pdf]

Genetics and Molecular Biology
https://doi.org/10.1590/1678-4685-GMB-2019-0392

Supplementary Material to "Characterization of three new mitochondrial genomes of Coraciiformes (*Megaceryle lugubris*, *Alcedo atthis*, *Halcyon smyrnensis*) and insights into their phylogenetics"

Table S6 - Nucleotide compositions in three codon positions of 12 mitochondrial protein-coding genes.

Species	1st codon position				2nd codon position				3rd codon position			
	A%	G%	C%	T%	A%	G%	C%	T%	A%	G%	C%	T%
Alcedo atthis	30.7	17.9	31.2	20.2	21.1	12.8	30.5	35.6	41.2	4.7	40.6	13.0
Ceryle rudis	31.5	19.4	29.2	20.1	20.3	12.6	29.6	37.7	41.2	3.6	42.2	12.5
Halcyon pileata	29.6	20.6	30.0	19.9	20.2	12.5	29.5	37.8	39.5	3.7	46.6	9.9
Halcyon smyrnensis	29.7	18.5	31.5	20.3	21.1	13.1	29.2	36.6	40.7	3.9	45.0	10.3
Halcyon coromanda	30.7	19.5	30.1	19.7	20.3	12.4	29.7	37.8	38.8	4.1	44.9	12.2
Megaceryle lugubris	30.7	17.5	31.3	20.8	21.3	12.7	29.6	36.5	37.7	5.2	39.3	17.8
Todirhamphus sanctus	30.3	19.5	30.6	19.7	20.3	12.7	29.1	38.0	37.8	5.5	43.4	13.1
Aceros waldeni	30.5	19.1	31.0	17.6	21.5	12.9	30.3	35.1	36.4	6.0	44.7	13.0
Bycanistes brevis	30.2	19.6	31.5	18.5	20.0	12.6	29.8	37.3	37.4	4.9	46.8	11.1
Penelopides panini	30.3	19.2	32.9	17.5	21.6	13.2	30.2	35.0	36.1	5.8	45.9	12.2
Eurystomus orientalis	30.4	19.6	30.0	20.1	20.3	12.4	29.7	37.7	38.8	4.7	42.7	13.8
Merops viridis	28.1	18.4	33.8	19.6	20.7	12.6	30.1	36.6	35.4	5.8	47.6	11.2
Upupa epops	31.3	19.5	28.7	20.5	20.1	12.5	29.8	37.6	42.4	4.7	39.9	13.0
Average	30.3	19.1	30.9	19.6	20.7	12.7	29.8	36.9	37.9	4.8	43.8	12.5
Range	3.4	3.1	5.1	3.3	1.6	0.8	1.4	3.0	7.0	2.4	8.3	7.9
