# Supplementary material for: Characterization of three new mitochondrial genomes of Coraciiformes (Megaceryle lugubris, Alcedo atthis, Halcyon smyrnensis) and insights into their phylogenetics
Source: Genet Mol Biol. 2020 Oct 5;43(4):e20190392. doi: 10.1590/1678-4685-GMB-2019-0392 (PMC7539371; doi:10.1590/1678-4685-GMB-2019-0392)
Supplement: Supplementary file 12 [file 1415-4757-GMB-43-4-e20190392-suppl12.pdf]

Genetics and Molecular Biology
https://doi.org/10.1590/1678-4685-GMB-2019-0392

Supplementary Material to "Characterization of three new mitochondrial genomes of Coraciiformes (*Megaceryle lugubris*, *Alcedo atthis*, *Halcyon smyrnensis*) and insights into their phylogenetics"

	160 … 170 … * … 180 … 190 … 199
A. atthis	ATT CGA TTT TTC CTC A GTA GCC ATT CTA TTC CTC CTA TTT
C. rudis	ATT CGA TTC TTC CTC - GTA GCC ATC CTA TTT CTC CTA TTC
H. pileate	ATC CGA TTT TTC CT- A GTA GCA ATC CTA TTC TTA CTA TTC
H. smyrnensis	ATC CGA TTT TTC CTC - GTA GCA ATC TTA TTC TTA CTG TTC
H. coromanda	ATT CGA TTT TTC CTC A GTA GCC ATT TTA TTC CTA CTA TTC
M. lugubris	ATT CGA TTC TTC CTC A GTA GCC ATC CTA TTC CTC CTA TTC
T. sanctus	ATC CGA TTC TTC CTC A GTA GCC ATC CTA TTC CTG CTG TTC
A. waldeni	GTA CGA TTC TTC CTC A GTA GCA ATC CTA TTC CTC CTA TTC
B. brevis	GTA CGA TTC TTC CTC A GTA GCA ATC CTA TTC CTC CTA TTC
P. panini	GTA CGA TTC TTC CT- A GTA GCA ATC CTA TTC CTC CTA TTC
E. orientalis	ATC CGA TTC TTC CT- C GTA GCT ATC CTA TTC CTA CTG TTC
M. viridis	ATC CGA TTC TTC CT- G GTA GCC ATC CTA TTC CTT CTA TTC
U. epops	ATC CGA TTT TTC CTC A GTA GCC ATC CTA TTC TTA CTA TTC

Figure S6 - Alignment of the mitochondrial MT-ND3 gene region spanning the extra base site (designated by *) for 13 species.
